# 

**Published:** 1864-04

**Authors:** 


					﻿Volume XXI.
Number V.
the Chicago
MEDICAL JOURNAL,
DeLASKIE MILLER, M. LI.
EPHRAIM INGALS, M. D.,
Kklitors and Proprietor**.
APRIL, 1864.
CHICAGO :
J. C. W. BAILEY, BOOK AND JOB PRINTER, 130 CLARK .STREET.
1804.
				

